# Structure of Urban Movements: Polycentric Activity and Entangled Hierarchical Flows

**DOI:** 10.1371/journal.pone.0015923

**Published:** 2011-01-07

**Authors:** Camille Roth, Soong Moon Kang, Michael Batty, Marc Barthélemy

**Affiliations:** 1 Centre d'Analyse et de Mathématique Sociales (CAMS), CNRS/EHESS, Paris, France; 2 Institut des Systèmes Complexes de Paris-Ile de France (ISC-PIF), Paris, France; 3 Department of Management Science and Innovation, University College London (UCL), London, United Kingdom; 4 Centre for Advanced Spatial Analysis (CASA), University College London (UCL), London, United Kingdom; 5 Institut de Physique Théorique, CEA, IPhT CNRS, URA 2306, Gif-sur-Yvette, France; University of Maribor, Slovenia

## Abstract

The spatial arrangement of urban hubs and centers and how individuals interact with these centers is a crucial problem with many applications ranging from urban planning to epidemiology. We utilize here in an unprecedented manner the large scale, real-time ‘Oyster’ card database of individual person movements in the London subway to reveal the structure and organization of the city. We show that patterns of intraurban movement are strongly heterogeneous in terms of volume, but not in terms of distance travelled, and that there is a polycentric structure composed of large flows organized around a limited number of activity centers. For smaller flows, the pattern of connections becomes richer and more complex and is not strictly hierarchical since it mixes different levels consisting of different orders of magnitude. This new understanding can shed light on the impact of new urban projects on the evolution of the polycentric configuration of a city and the dense structure of its centers and it provides an initial approach to modeling flows in an urban system.

## Introduction

The structure of a large city is probably one of the most complex spatial system that we can encounter. It is made of a large number of diverse components connected by different transportation and distribution networks. In this respect, the popular conception of a city with one center and pendular movements going in and out of the business center is likely to be an audacious simplification of what actually happens. The most prominent and visible effects of such spatial organization of economic activity in large and densely populated urban areas are characterized by severe traffic congestion, uncontrolled urban sprawl of such cities and the strong possibilities of rapidly spreading viruses, biologial and social, through the dense underlying networks [1]–[3]. The mitigation of these undesirable effects depends intrinsically on our understanding of urban structure [4], the spatial arrangement of urban hubs and centers, and how the individuals interact with these centers. The dominant model of the industrial city is based on a monocentric structure [5], [6], but contemporary cities are more complex, displaying patterns of polycentricity that require a clear typology for their understanding [7]. One of the most important features of an urban landscape is the clustering of economic activity in many centers [8]: the idea of the polycentric city in such terms can be traced back over one hundred years [9], [10], but so far no clear quantitative definition has been proposed, apart from various methods of density thresholding based, for example, on employment [11]. In order to characterize polycentricity, we must investigate movement data such as person flow and mobile-phone usage [12] which offers the possibility of analyzing quantitatively various features of the spatial organization associated with individual traffic movements. More precisely, in this study, we analyze data for the London underground rail (‘tube’) system collected from the Oyster card (an electronic ticketing system used to record public transport passenger movements and fare tariffs within Greater London) which enables us to infer the statistical properties of individual movement patterns in a large urban setting.

## Results

World cities [13] are among those with the most complex spatial structure. The number, the diversity of components and their localization warns us intuitively that these megapoles are far from their original historical form which is invariably represented by a simple, monocentric structure. In particular, the level of commercial and industrial activity varies strongly from one area to another. Thus flows of individuals can be thought as good proxies for the activity of an area and to this end we first checked that the flows at different stations correlate positively with other activity indicators such as counts of employees and the employee density. This shows that indicators of a different nature and on different time scales, which are also widely regarded as measures of polycentricity in large cities, are also consistent with movement data recorded over much shorter time scales.

The main results that we will discuss in this section are that (i) flows are generally of a local nature (ii) they are also organized/aggregated around polycenters and (iii) the examination and decomposition of these flows lead to the description of entangled hierarchies, and (iv) hence one likely structure describing this large metropolitan area is based on polycentrism. This perspective thus draws new insights from data that has become available from electronic sources that have so far not been utilised in analyzing the urban spatial structure and in this sense, are unprecedented in the field.

**Figure 1:**
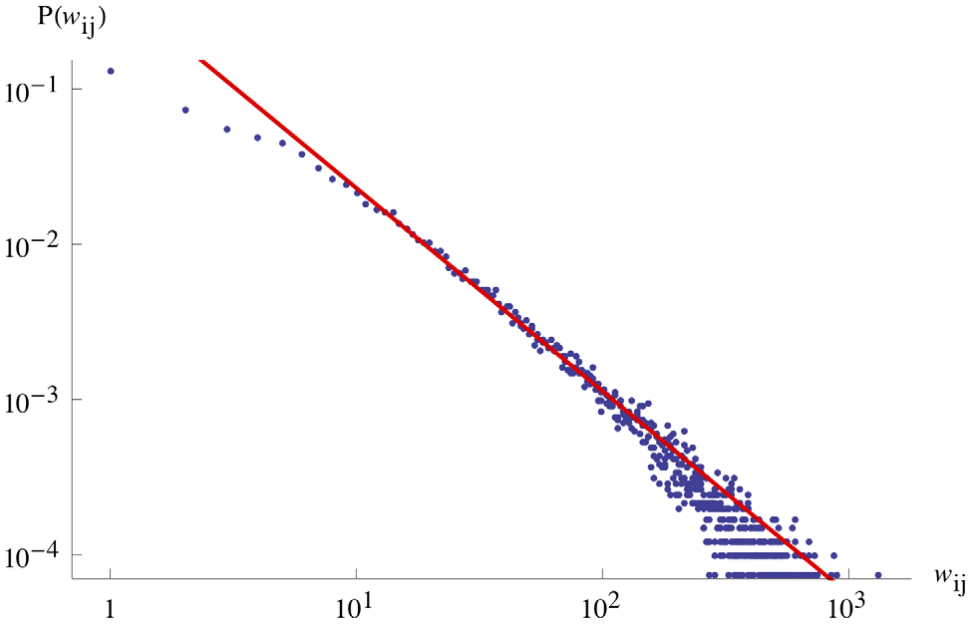
Flow distribution. Loglog plot of the histogram of the number of trips between two stations of the tube system. The line is a power law fit with exponent 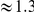.

To get a preliminary grasp on the data, we observe that the flow distribution (normalized histogram of flows of individuals) is fitted by a power law with exponent 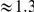 which indicates that there is strong heterogeneity of individuals' movements in this city (for this distribution, the ratio of the two first moments has a large value 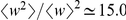, which confirms this strong heterogeneity)— see Figure 1. Broad distribution of flows have already been observed at the inter-urban level [14], but it is the first time that we observe this empirically at an intra-urban level showing that, in agreement with other studies (for Madrid [15] and for Portland, Oregon [1]), the movement patterns in large cities exhibit an heterogeneous organization of flows.

**Figure 2:**
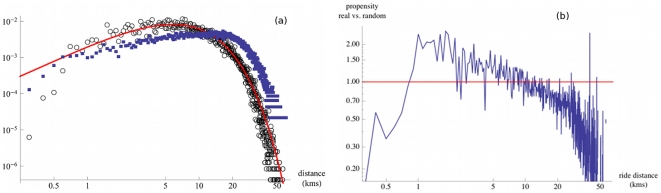
Ride distance distribution and propensity. (a) Superimposition of the distance distribution of rides (circles) and of the distance distribution between stations (squares). The distribution of the observed rides can be fitted by a negative binomial law of parameters 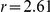 and 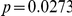, corresponding to a mean 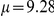
kms and standard deviation 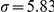
kms (solid line). This distribution is not a broad law (such as a Levy flight for example), in contrast to previous findings using indirect measures of movement [12], [16]. (b) Ride distance propensity. Propensity of achieving a ride at a given distance with respect to a null-model of randomized rides.

Spatial separation is another primary feature of movement and we show in Figure 2*a* the raw distribution of rides occurring between two stations at a given distance. This distribution can be fitted by a negative binomial law rather than a broad law such as the Levy flights suggested in [12], [16].

While this graph exhibits actual commuting patterns, it does not tell us much about commuter behavior, all other things being equal. Indeed, the geographical constraints are important and the distance distribution between stations (shown superimposed in Figure 2*a*) could be a major factor in the ride distribution. Also, the particular flow distribution over the network is likely to bias the ride distance distribution: rides corresponding to two stations, which have respectively a large outflow and inflow, should be more likely, hence the distance between these two stations is likely to be overrepresented in the previous distribution. This bias relates to how much agents prefer to use the underground to achieve rides at a given distance. In order to estimate the part governed by the individuals behavior, we use a null-model for randomizing rides in such a way that total outflows and total inflows at each station are conserved while actual ride extremities are reshuffled (see Methods). Put differently, the random null-model corresponds to a flow matrix that should normally occur given particular out- and inflows at stations, irrespective of agent's preferences. Dividing the real-world values by the random flow matrix (averaged over 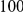 random simulations) gives the propensity (see Methods) which is an estimate of how much the real data deviates from a random setting. Results are described in Figure 2*b*. We observe that rides covering a distance of around 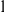 to 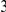
kms are twice as likely. The propensity continuously falls to 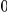 for longer rides, and is significantly less than one for rides of less than 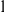
km. Above a distance of 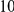
kms, the propensity is less than one indicating that individuals are less inclined to use the subway for longer distances. Hence, all other things being equal, people are less inclined to take the tube for rides not covering this sort of ‘typical’ distance.

**Figure 3:**
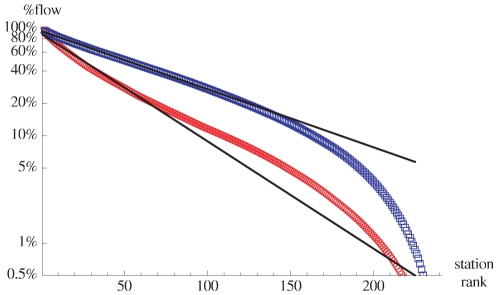
Total flow distributions. Zipf plot for the total inflows (*red circles*, *below*) and total outflows (*blue squares*, *above*) for morning peak hours (7am–10am). The inflow 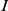 (outflow 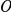) of a station 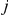 (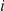) is defined as 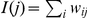 (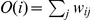). The straight lines are exponential fits of the form 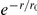 with 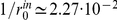 for the inflow and 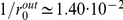 for the outflow.

In addition to being strongly heterogenous, rides are therefore to some extent essentially local. At a more aggregated level, and in order to infer the city structure at a larger scale, we can study the distribution of incoming (or outgoing) flows for a given station. We show in the Figure 3 the rank-ordered total flows (Zipf plots) for the morning peak hours on a lin-log graph displaying an exponential decay (Flows for evening peak hours (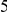
pm–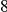
pm) reveal a roughly inverse pattern, i.e. the total outflow is concentrated on a few centers, and similarly but less markedly, the same occurs for total inflows).

The exponential decay of these plots demonstrate that most of the total flows are concentrated on a few stations. Indeed, an exponential decay of the form 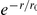, where 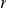 is the rank, is a signature of the existence of a scale 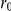. In this case, the exponential fit shows that the number of important inflow stations is of order 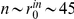 and larger for outflow stations. During the morning peak hours, essentially, stations that generate a large inflow have a smaller outflow, and vice-versa. Also, rides are statistically balanced over the entire day, which suggests that rides are essentially round trips. From this analysis, we can conclude that the activity is concentrated in a small number of centers dispersed over the city. Using the exponential distribution of flows, we can then define multiple centers acting as sources or sinks depending on the time of day.

**Figure 4:**
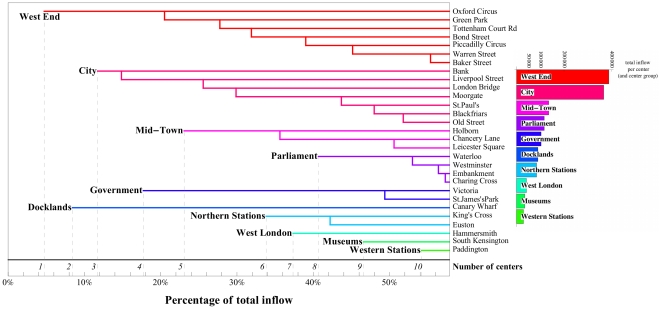
Hierarchical organization of the activity: Polycenters. Breakdown of centers in terms of underlying stations and inflows. We gather stations by descending order of total inflow and we aggregate the stations to centers when taking into account more and more stations. In this process, all stations within 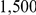 meters of an already-defined center are aggregated to this main center. This yields the dendrogram shown here which highlights the hierarchical nature of the polycentric organization of this urban system. The bold names to the left of the aggregates — such as “*West End*” for the group of stations around Oxford Circus — are used throughout the paper as convenient labels to denote the polycenters.

To examine further this polycentric structure, we will aggregate different stations if their inflow is large and they are spatially close to one another. Various clustering methods could be used and we choose one of the simplest described in the section Methods. This clustering yields a hierarchical, descending decomposition of inflows with respect to an increasing share of the total inflow in the network. We summarize the results of this process in the dendrogram shown in Figure 4. This dendrogram highlights the hierarchical organization of urban polycentricity. The number of centers is not an absolute quantity, but depends on an observation scale as measured here by the percentage of inflow. As we consider higher percentages of the total inflow, more centers are taken into account, which leads to centers as an aggregate of multiple sub-centers with smaller inflows. In other words, this is equivalent to saying that at large spatial scales, we observe one large center corresponding to the whole city, and when we decrease the scale of observation, multiple centers appear, which are themselves composed of smaller centers. This hierarchical nature is crucial and indicates that we cannot define a center by applying a threshold rule (e.g., an area is a center if the population or employment density is larger than some threshold [11]), but that it can only be defined according to a given scale.

**Figure 5:**
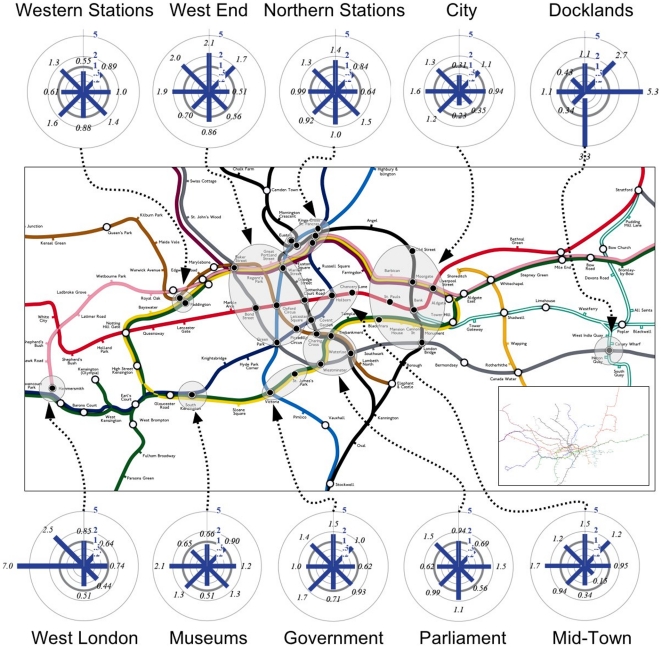
The London subway (tube) system: polycenters and basins of attraction. In the inset, we show the entire tube network while in the main figure, we zoom in on the central part of London. We represent the ten most important polycenters defined in the dendrogram of Figure 3, and show the corresponding propensity to anisotropy comparing actual flows with the null model defined in the text. A propensity of 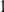 means that there is no deviation in a given direction with respect to the null model. Circles correspond to various levels of identical propensity values: the thicker circle in the middle corresponds to 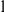, inner circles correspond to propensities of 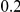 and 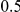, and outer circles to 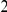 and 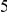. The anisotropy is essentially in opposite directions from the center, thus showing a strong bias towards the suburbs for peripheral centers essentially, rather than for central centers. Moreover, most stations control their own regions and seem to have their own distinctive basins of attraction.

We represent the ten most important polycenters defined in the dendrogram of Figure 4, and show the corresponding propensity to anisotropy comparing actual flows with the null model defined above (see Methods). This comparison shows that the actual flows are in general very different from what is obtained using the random null model. We study the relative orientation of the incoming flow (normalized by its corresponding quantity given by the null model) and picture it by eight-segment compasses, which we show in Figure 5 on the central and inner London underground map. The absence of any bias would give a fully isotropic compass with all segments of radius equal to one (propensity equal to 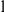). The anisotropy is essentially in opposite directions from the center, thus showing a strong bias towards the suburbs essentially for peripheral rather than for central centers.

We now examine how the flows are distributed into and outside centers, focusing on the morning peak hours. We first aggregate the flows by centers by computing the total flow incoming to a certain center 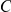:
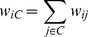
(1)In this aggregated view, we thus represent movements by a directed network where flows go from single stations (the sources) to centers, which are groups of stations.

**Figure 6:**
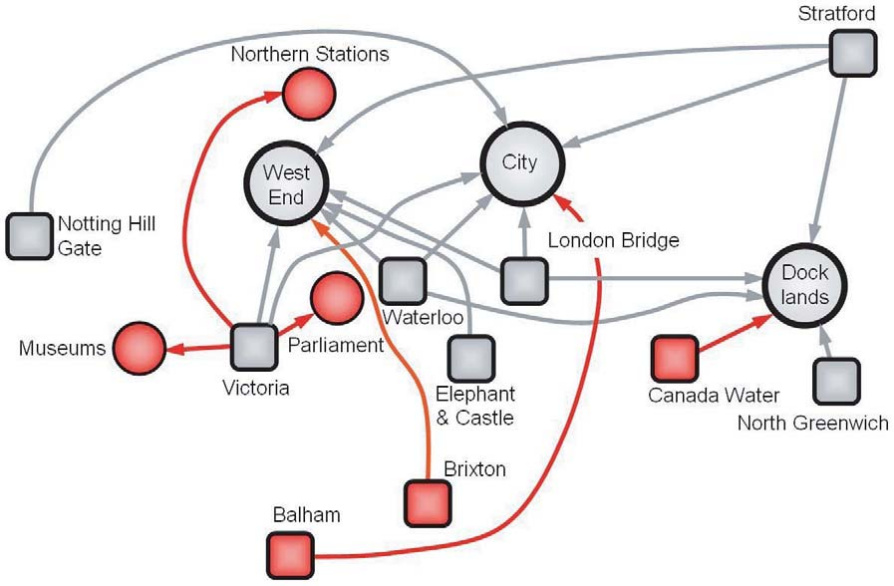
Structure of flows at 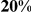 and 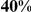 of the total flow. When considering the most important flows from stations to centers such their sum represents 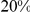 of the total flow in the network, we observe sources (represented as squares) with outdegree 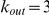 such as London Bridge, Stratford, or Waterloo connecting to three different centers (represented as circles), as well as sources with 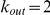 (eg. Victoria) and 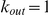 (eg. Elephant and Castle). We also show how the pattern of flows is constructed iteratively when we go to larger fraction of the total flow (from 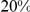 shown in black to 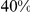 shown in red). We represent in red the new sources, centers and connections. The new sources connect to the older centers (eg. *West End*, *City*, etc) and the existing sources (eg. *Victoria*) connect to new centers (eg. *Northern stations*, *Museums*, and *Parliament*).

We then rank all flows 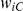 in a decreasing order, thereby focusing on paths of decreasing importance as if we were detailing a map starting with highways, then concentrating on roads, and then on streets. We consider the 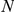 most important flows such that the corresponding sum of flows is a given percentage 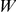 of the total flow in the network. For example, if we consider the flows up to 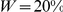 of the total flow, we obtain the structure that we show in Figure 6 (it should be noted that we kept the ‘station-to-center’ flows such that they represent 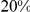 of the total flow, which is different from keeping the most important station-to-station flows such as it is done for the Figure 4 precisely in order to define those ‘centers’. We thus cannot directly compare these Figures 4 and 6).

At this scale, it is clear that we have three main centers and sources (with various outdegree values), which mostly correspond to intermodal rail-subway connections. Adding more links, we reach a fraction 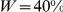 of the total flow and we then investigate smaller flows at a finer scale. We see that we have new sources appearing at this level and new connections from sources that were present at 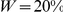.

**Figure 7:**
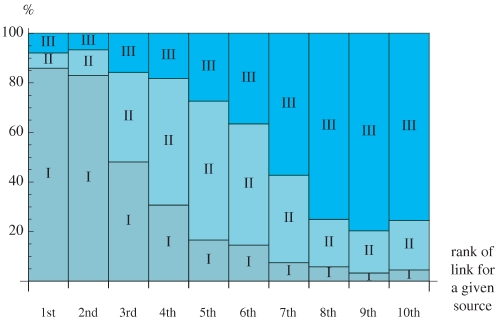
Most important links. Proportion of links going from sources to centers of a certain group (I, II, III), considering links of decreasing importance for each given source, when raising 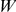 (from the first link appearing, at left, to the last link, at right).

We can summarize this result with the graph shown in Figure 7 where we divide the centers into three groups according to their inflow (decreasing from first Group I to the last Group III). In other words (see Figure 4), Group I gathers centers with the most important *total* inflow namely the *West End*, *City* and *Mid-town*. Group II gathers the next three centers *Parliament*, *Government* and *Docklands* while Group III gathers the other centers such as the *Northern stations*, *West London*, *Museums* and the *Western stations*. This figure shows that for more than 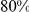 of the sources, the most important link (ie. the 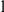
st link) connects to a center of Group I. Conversely for more than 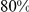 of the sources, the least important link (ie. 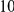
th link) goes to a center of Group III. The flow structure thus follows an original yet simple pattern when we explore smaller and smaller weights.

We can quantify in a more precise way how the structure of flows evolves when we investigate smaller flows by exploring the list of flows 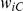 in decreasing order and by introducing the transition matrix 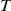, which describes how the outdegree of a source varies with increasing 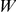 (see Methods). When we explore smaller flows, the analysis of the T-matrix shows that the pattern of connections from sources to centers becomes richer and more complex, but can nonetheless be described by the simple iterative process described above: the most important link of a source goes to the most important centers, the second most important link connects to the second most important centers, and so on. It is interesting to note that even if the organization of flows follows a simple iterative scheme, it leads to a complex and rich structure, which is not strictly hierarchical since it mixes different levels of flows consisting of different orders of magnitude. In addition, the fact that the most important flows always connect to the same center naturally leads to the question of efficiency and congestion in such a system. In this respect, London appears as a ‘natural’ city as opposed to an ‘artificial’ city for which flows would be constructed according to an optimized, hierarchical schema [17], [18].

## Discussion

World cities such as London have tended to defy understanding hitherto because simple hierarchical subdivision has ignored the fact that their polycentricity subsumes a pattern of nested urban movements. Using the Oyster data we can identify multiple centers in London, then describe the traffic flowing into these centers as a simple hierarchic decomposition of multiple flows at various scales. In other words, these movements define a series of subcenters at different levels where the complex pattern of flows can be unpacked using our simple iterative scheme based on the representation of ever finer scales defined by smaller weights. Casual observation suggests that this kind of complexity might apply to other world cities such as Paris, New York or Tokyo where spatial structure tends to reveal patterns of polycentricity considerably more intricate than cities lower down the city size hierarchy. Our approach needs to be extended of course to other modes of travel, which will complement and enrich the analysis of polycentricity. The Oyster card is already used on buses and has just expanded beyond the tube system to cover other modes of travel such as surface rail in Greater London. With GPS traffic systems monitoring, in time, all such movements will be captured, extending our ability to understand and plan for the complexity that defines the contemporary city.

## Methods

### Material

Our analysis of individual movements is based on a dataset describing the entire underground service between 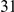 March 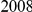 and 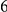 April 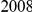 encompassing a total of 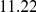 million trips from 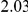 million individual Oyster card IDs. For each trip, the data includes the origin and destination for individual passengers as well as the corresponding time of the trip. We stress that the data we obtained from Transport for London (TfL) is completely anonymized without any possibility of trace back to individuals. Besides, we only have individual trajectories, but not the history of the trajectories over a long period of time which then could provide the capability of identifying individuals from the electoral register and business directories. From this dataset, we build the (origin/destination) flow matrix 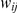, which gathers the aggregated number of rides leaving a station 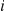 to a station 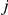 over a given period of time. The analysis of these flow matrices in several time intervals for every single day in the dataset shows that the commuting patterns during weekdays present a regular and distinctive pattern in contrast to travel at weekends. As a result, we focus our study on the commuting patterns during weekdays.

### The null model, propensity, and anisotropy

#### The null model

The subway infrastructure imposes a certain number of physical constraints which can affect various distributions. This is for example the case of the ride distribution where rides between two stations with large outflow and inflow, respectively, are likely to be over-represented. As such the ride distribution could simply be a result of the peculiar subway spatial structure. In order to eliminate this type of biases, we use for comparison a null-model constructed in the following way. We randomize rides in a such a way that the total outflow and total inflow of each station is conserved while actual ride extremities are reshuffled. This model is basically a configuration model [19], [20] which preserves the total number of incoming and outgoing links for each station and where each link corresponds to a given ride. Put differently, the random setting corresponds to a flow matrix (obtained here by an average over 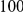 random simulations) that should normally occur given particular out- and in-flow heterogeneity at stations, irrespective of agent preferences.

#### The ride propensity

We can then divide the real values of flows 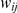 by the random flow matrix which yields an estimate of how much the real data deviates from a random setting (at fixed inflow-outflow constraints). For the ride distribution we then obtain the *ride propensity* 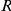 shown in Figure 2*b*

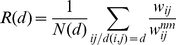
(2)where 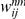 is the number of individuals going from 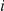 to 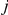 in the null model, 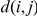 represents the distance on the network between 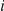 and 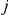, and where 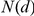 is the number of pairs of nodes at distance 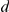. This propensity gives an estimate of how much the real data deviates from a random flow assignment with the same geographical and flow constraints. In other words, when the propensity is equal to one the observed flows are entirely due to the geographical and flow structure of the network. Conversely when the propensity is smaller or larger than 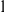, the flows reflect non-uniform preferences for rides of certain distance.

#### The anisotropy propensity

We used the null model in order to extract the part due to the behavior of the commuters in their ride distribution. We can also study the relative orientation of the incoming flow normalized by its corresponding quantity given by the null model which gives the anisotropy 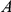 due to the commuters behavior
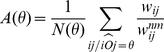
(3)where 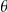 is a particular direction (we binned the angle in eight equal intervals so to represent an eight-segment compass) and where the sum is over the 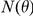 nodes 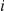 and 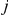 such that the angle of 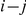 is given by 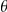. The absence of any bias would give a fully isotropic compass with all segments of radius equal to one (anisotropy propensity equal to 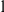).

### Identifying the polycenters

Clustering methods for point in spaces has been the subject of many studies and are used in many different fields. In particular, in computational biology and bioinformatics, clustering is used to build group of genes with related expression patterns. Many different methods were developed and the most common ones are hierarchical clustering methods (such as those based on K-means and their derivatives, see for example [21]). Here, we are in a slightly different position. The stations are clearly located in space and thus Euclidean distance appears as the natural distance measure (a necessary ingredient for clustering methods). Yet these stations are also characterized by their inflow. For this reason, the usual methods are not directly applicable and we thus adopted the simplest clustering method which we describe as follows. We first gather stations by descending order of total inflow, thereby defining centers of decreasing importance. In order to account for geographical proximity of groups of stations, indicating subsets of distinct stations belonging to a single geographical center, we aggregate all stations within a distance 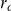 of an already-defined center. In this way we systematically increase the total flow associated with these centers and we continue this process until we capture a large percentage of the total flow. We thus chose to stop at 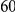 percent of the total flow in order to avoid to include too many details and too much noise.

We varied the value of 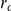 from 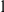 to 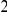 kms and observed that our results were stable. This stability probably comes from the fact that the inter-distance station is of order 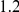
kms for London in 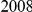 and corresponds to some psychological threshold above which individuals prefer to take the subway if they can choose. The results discussed above are obtained with 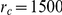 meters.

### The T matrix

We face here a difficult problem: we have a complete weighted directed network featuring flows from stations to centers, and the goal is to extract some meaningful information. We started with the analysis of the dominant flows and we would like to understand how the flows are structured when we explore smaller values. In order to do this, we introduce a ‘transition’ matrix 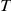 which characterizes quantitatively the changes in the flow structure when we explore the list of flows 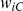 going from a station 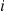 to a center 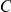 in decreasing order of importance. In what follows, when we talk of ‘total flow at 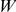
’, we mean that we consider only the most important flows 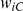 so that we reach a total fraction 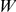 of the total flow on the whole network of station-to-center flows. When the total flow goes from 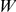 to 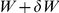, the elements 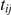 of 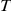 represent the number of sources with outdegree 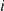 at 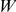 and with outdegree 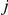 at 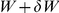. Note that 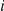 starts at 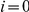 while 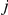 starts at 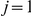 (i.e. 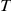 only denotes sources that have a strictly positive outdegree at 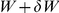).

As an example, when we go from 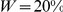 to 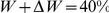, the 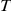 matrix is
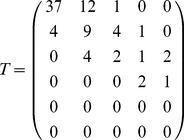
(4)


**Figure 8:**
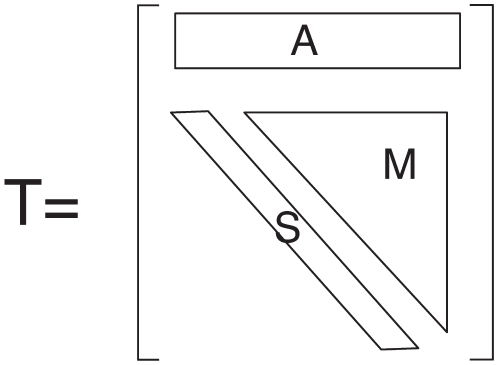
Transition matrix. Typical form of the outdegree transition matrix 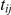, consisting essentially of a row vector (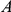, inexistent sources before the transition) and an upper triangular matrix (made of a diagonal 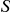 of sources having the same out-degree after the transition, and a submatrix 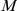 of sources whose out-degree increases after the transition).

The matrix 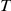 is composed of three parts (see Figure 8). The first part, 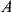, consists of new sources appearing when we increase the total flow, and corresponds to the first line of 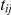 where 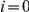. The second part, 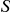, consists of sources where the outdegree stays invariant when we change from 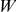 to 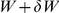 (i.e., the diagonal 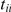). The third part, 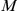, consists of sources that were already present at the 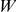 level and the outdegree changes during the process from 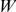 to 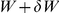 (i.e., the upper triangle 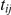 where 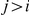). We can compute the number of sources in each of these types and plot them. A proper 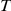 matrix is a 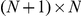 matrix (in Eq. 4, 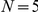), as the 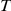 matrix is made of a row vector (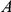) and an upper triangular matrix (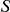, 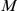 and the zeros) because a source that feeds 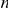 centers cannot become a source feeding 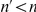 centers when transitioning to a larger inflow-cut 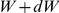. The row vector 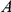 indicates sources that were not feeding centers before, and now feed some centers, i.e., sources that were non-existent for a lower inflow-cut, hence the extra initial row represented by vector 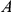. Thus, ‘
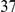
’ means that after the transition (at the new inflow-cut), there are 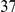 new sources feeding one center, 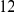 new sources feeding two, 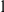 new source feeding three. The ‘
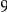
’ on the second row means that 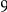 sources that used to feed one center, now feed two, and so on. The row 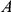 is thus given by
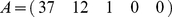
(5)and the diagonal is
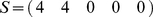
(6)The upper triangular matrix 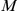 is given by
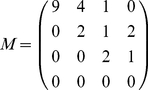
(7)


In the case of the transition 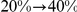, the major phenomenon is the appearance of new sources (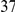 in this case) followed by sources feeding new centers.

**Figure 9:**
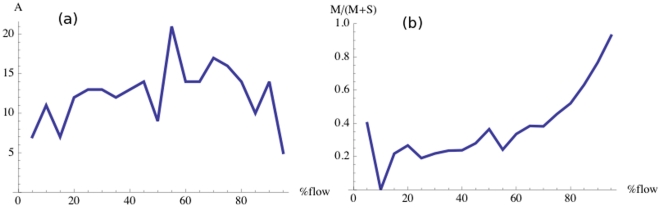
Evolution of the number of sources and their type. (a) Number of new sources (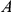) versus the total flow 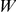. (b) Fraction of existing sources whose type is changing (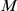) when the total flow varies from 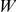 to 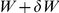. Here 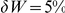.

Figure 9a shows the number of new sources (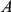 in the matrix 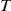) and the sources that change type (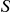). We observe that there is a continuous addition of new sources along with connections to new and old centers. Besides, for a total flow less than 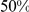, there is a relatively stable proportion of sources (about 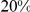) whose outdegree varies when 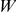 increases. When we zoom into finer scales (i.e., larger values of the total flow 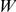), new sources appear and connect preferentially to the existing largest centers, while the existing sources connect to the new centers through secondary connections. This yields two types of connection only. The first type goes from new sources to old centers, and the second type from old sources to new centers.
